# Random Mutagenesis Applied to Reveal Factors Involved in Oxidative Tolerance and Biofilm Formation in Foodborne *Cronobacter malonaticus*

**DOI:** 10.3389/fmicb.2019.00877

**Published:** 2019-05-01

**Authors:** Maofeng Zhang, Xiyan Zhang, Liaowang Tong, Dexin Ou, Yaping Wang, Jumei Zhang, Qingping Wu, Yingwang Ye

**Affiliations:** ^1^School of Food Science and Engineering, Hefei University of Technology, Hefei, China; ^2^State Key Laboratory of Applied Microbiology Southern China, Guangdong Provincial Key Laboratory of Microbial Culture Collection and Application, Guangdong Institute of Microbiology, Guangzhou, China

**Keywords:** *Cronobacter malonaticus*, random mutagenesis, arbitrary PCR, oxidative stress, biofilm formation

## Abstract

*Cronobacter* species are linked with life-treating diseases in neonates and show strong tolerances to environmental stress. However, the information about factors involved in oxidative tolerance in *Cronobacter* remains elusive. Here, factors involved in oxidative tolerance in *C. malonaticus* were identified using a transposon mutagenesis. Eight mutants were successfully screened based on a comparison of the growth of strains from mutant library (*n* = 215) and wild type (WT) strain under 1.0 mM H_2_O_2_. Mutating sites including thioredoxin 2, glutaredoxin 3, pantothenate kinase, serine/threonine protein kinase, pyruvate kinase, phospholipase A, ferrous iron transport protein A, and alanine racemase 2 were successfully identified by arbitrary PCR and sequencing alignment. Furthermore, the comparison about quantity and structure of biofilms formation among eight mutants and WT was determined using crystal violet staining (CVS), scanning electron microscopy (SEM), and confocal laser scanning microscopy (CLSM). Results showed that the biofilms of eight mutants significantly decreased within 48 h compared to that of WT, suggesting that mutating genes play important roles in biofilm formation under oxidative stress. The findings provide valuable information for deeply understanding molecular mechanism about oxidative tolerance of *C. malonaticus*.

## Introduction

*Cronobacter* species are important foodborne pathogens causing life-threating infections in infants (Van Acker et al., 2001; Healy et al., 2010). Contaminated powdered infant formula (PIF) is considered to be the major transmission route of *Cronobacter* infections (Biering et al., 1989; Van Acker et al., 2001; Norberg et al., 2012; Ye et al., 2014). So, the high risks of *Cronobacter* strains in powdered infant formula on newborn’s health has arouse public concerns.

*Cronobacter* spp. show unusual abilities to survive under environmental stress (Gurtler et al., 2005). To date, the genus of *Cronobacter* includes *C. sakazakii*, *C. malonaticus*, *C. turicensis*, *C. muytjensii*, *C. dublinensis*, *C. universalis*, and *C. condiment* (Iversen et al., 2008). The factors involved in oxidative stress in *C. sakazakii* have been reported. For example, polymorphisms in RpoS sequence and Significant heterogeneity of stress tolerance including oxidative stress among natural isolates of *C. sakazakii* has been described (Alvarez-Ordóñez et al., 2012). Johler et al. (2010) demonstrated that genes including *crtX*, *crtE*, and *crtY* involved in yellow pigmenting of *C. sakazakii* ES5 affected tolerance to oxidative stress. In *C. sakazkaii* ATCC29544, Hfq, an RNA chaperone, has been found to increase the tolerance to oxidative stress (Kim et al., 2015). *C. malonaticus* has been implicated in infections in infant and adults (Forsythe et al., 2014; Alsonosi et al., 2015). PIF is the major source of *C. malonaticus* (Ogrodzki and Forsythe, 2015, 2017). Hydrogen peroxide (H_2_O_2_) is a well-studied sanitizer for inactivate foodborne pathogens. In addition, Ye et al. (2018) determined the inhibitory effects of H_2_O_2_ on *C. malonaticus* cells and its biofilm formation. However, information about factors involved in oxidative tolerance in *C. malonaticus* is largely unknown.

In this study, a transposon mutagenesis approach was applied to reveal the factors involved in resistance to oxidative stress, and the biofilm formation among mutants and WT strains were further detected using crystal violet staining (CVS), scanning electron microscopy (SEM), and confocal laser scanning microscopy (CLSM) to reveal potential relationship between oxidative stress and biofilm formation.

## Materials and Methods

### The Development of Mutants Library

The procedure of transposon mutagenesis approach was performed as described by Zhang et al. (2018).

### Screening of Mutants Tolerance to Oxidative Stress

For screening positive mutants tolerant to oxidative stress, overnight culture (OD_600_ = 0.8, v/v, 1%) was inoculated into LB broth (Luqiao, Beijing) with 1.0 mM H_2_O_2_ at 37°C for 8 h. Growth of mutants (*n* = 215) and WT strain were measured spectrophotometrically in 96-well culture plates (Corning, New York, NY, United States) by determining the optical density at 600 nm (OD_600_). Each experiment was independently done in triplicate. Growth of strains were analyzed by the statistical analysis of *t*-tests using OriginPro 8.5.1 software. A significant difference was defined as a *p*-value (*p* < 0.05) between wild-type (WT) and mutants.

### Identification of Mutating Sites

The detailed procedure for identification of mutating sites and analysis of inserting sites was performed as described by Zhang et al. (2018). In brief, the mutating genes were amplified by arbitrary PCR, then the fragments were purified for being sequenced and aligned.

### Comparison of Biofilm Formation Among Mutants and Wild Type

Under oxidative stress (LB with 1.0 mM H_2_O_2_), biofilm formation using CVS was determined ranging from 24 to 72 h described previously by Zhang et al. (2018). In addition, the biofilms on the cell slips at 48 h was detected using SEM (Hitachi, Tokyo, Japan) and CLSM (Zeiss, Berlin, Germany) using LIVE/DEAD BacLight bacterial viability Kit according to instructions (Invitrogen, Carlsbad, CA, United States).

## Results and Discussion

**FIGURE 1:**
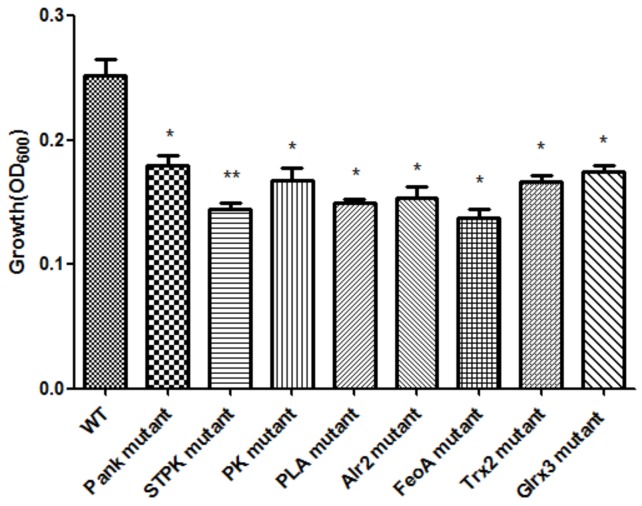
Growth of *C. malonaticus* wild strain (WT) and mutants under 1.0 mM H_2_O_2_ oxidative stress. Bar = mean ± SD. ^∗^Significant difference about growth under 1.0 mM H_2_0_2_ with WT strain (*p* < 0.05), ^∗∗^significant difference about growth under 1.0 mM H_2_0_2_ with WT strain (*p* < 0.01).

Based on the growth of mutants and WT strain under oxidative stress (1.0 mM H_2_O_2_), eight mutants were successfully screened, and the growth of eight mutants under oxidative stress was significantly (*p* < 0.05) decreased compared with that of WT shown in Figure 1. The mutating genes listed in Table 1 encode thioredoxin 2 (Trx2), glutaredoxin 3 (Grx3), pantothenate kinase (Pank), serine/threonine protein kinase (STPK), pyruvate kinase (PK), phospholipase A (PLA), ferrous iron transport protein A (FeoA), and alanine racemase 2 (Alr2) which contributed to tolerance to oxidative stress in *C. malonaticus*.

**Table 1:** Transposon insertion sites involved in oxidative stress in *C. malonaticus* YE01.

Mutant	Gene function of	Relevant	Accession
strains	encoded protein	features	No.
Trx2 mutant	Thioredoxin 2	The Component of TrxR	AHB69350
Glrx3 mutant	Glutaredoxin 3	Participate in the redox reaction	AHB72372
Pank mutant	Pantothenate kinase	The synthesis of CoA	AHB72217
STPK mutant	Serine/threonine protein kinase	Stimulate production of proteins of serine/thronine	AHB68419
PK mutant	Pyruvate kinase	The synthesis of pyruvate	AHB70675
PLA mutant	Phospholipase A	Hydrolyzed glycerin phospholipids	AHB72248
FeoA mutant	Ferrous iron transport protein A	Transport iron	AHB68639
Alr2 mutant	Alanine racemase 2	The transformation of alanine isomers	AHB70086

In *Escherichia coli*, thioredoxin 2 (encoded by *trxC*) was identified on the basis of sequence similarity (Miranda-Vizuete et al., 1997), but *trxC* mutants do not show altered sensitivity to H_2_O_2_ (Ritz et al., 2000). In addition, inactivity of thioredoxin 1 (encoded by *trxA*) and thioredoxin reductase (encoded by *trxB*) caused more sensitive to H_2_O_2_ in stationary phase of *E. coli* (Takemoto et al., 1998). Glutaredoxin (Grx) is a thiol-disulfide oxidoreductase widely distributed from bacteria to higher eukaryotes (Rouhier et al., 2008). In yeast, mutants lacking Grx are sensitive to oxidative stress (Luikenhuis et al., 1998). The OxyR and SoxR in *E. coli*, and the *S. cerevisiae* Yap1p transcriptional regulators were modulated by glutathione- and thioredoxin-dependent reduction systems for the adaptive responses to oxidative stress (Carmel-Harel and Storz, 2000). The inactivity of glutaredoxin 2 and glutaredoxin 3 encoded by *grxB* and *grxC*, respectively, were found in *E. coli* strains lacking glutaredoxin 1 and thioredoxin 1 still showed GSH oxidoreductase activity (Aslund et al., 1994). The inactivity of glutaredoxin 2 in *E. coli* cells were more sensitive to hydrogen peroxide and other oxidants, and the interconnection between catalases and thioredoxin/glutaredoxin pathways in the antioxidant response was observed (Vlamis-Gardikas et al., 2002). Regulators including OxyR, SoxRS, and RpoS in *E. coli* were associated with the tolerance to oxidative stress (Chiang and Schellhorn, 2012). The redox proteins such as Grx A (Grx1) required for maintaining redox status in bacteria also protect bacteria from oxidative stress (Caldas et al., 2006; Meyer et al., 2009). The pantothenate kinase is required for the biosynthesis of coenzyme A (CoA). In *Bacillus anthracis*, the type III pantothenate kinase plays important roles in maintenance of cytosolic redox balance and in adaptation to the oxidative stress in *B. anthracis* (Paige et al., 2008).

Ferrous iron (Fe^2+^) is one of the essential elements required for growth and virulence of the majority of pathogens (Hayrapetyan et al., 2016). Here, ferrous iron transport contributed to oxidative tolerance in *C. malonaticus* through the reduction reaction of Fe^2+^ to attenuate the injuries from oxidation (H_2_O_2_). The ferrous iron transport (*feo*) operon was first discovered in *E. coli* K12 in 1987 through studies of a series of ferrous iron transport mutants, and the deletion of *feo* strains cause the failure to taking up ferrous iron (Hantke, 1987). In addition, in the absence of FeoB, *H. pylori* was unable to colonize the gastric mucosa of mice (Velayudhan et al., 2000). Naikare et al. (2006) found that FeoB is essential for the uptake of ferrous iron, gut colonization and intracellular survival. On the Contrary, *feo* deletions in *V. cholerae* do not seem to affect its colonization in the mouse model (Wyckoff et al., 2006).

Through 2-D method combined with MALDI-TOF-MS and database queries, pyruvate kinase was involved in enhancement of oxidative stress in *Pichia caribbica* (Zhang et al., 2017). In the mitochondrial, pyruvate kinase M2 isoform (PKM2) regulates oxidative stress-induced apoptosis by stabilizing B-cell lymphoma 2 (Bcl2) (Liang et al., 2017). Brien et al. reported that increased placental phospholipase A2 gene expression was implicated in oxidative stress in preeclampsia (Brien et al., 2017). Expression of serine/threonine protein kinase and peroxisomal catalase in *P. caribbica* were involved in the enhancement of oxidative stress tolerance and biocontrol efficacy of *P. caribbica* (Zhang et al., 2017). Serine/Threonine kinases activation was induced by oxidative stress in frontotemporal dementia (Palluzzi et al., 2017). *S. mutans* expresses a eukaryotic serine/threonine type kinase known as STPK which enhances resistance to oxidative stress (Zhu and Kreth, 2010). Likewise, our results also found that inactivity of pantothenate kinase (Pank), serine/threonine protein kinase (STPK), pyruvate kinase (PK) caused sensitive to oxidative stress. To date, roles of Phospholipases (PLs) on tolerance to oxidative stress are not reported in other foodborne pathogens except for *C. malonaticus*.

**FIGURE 2:**
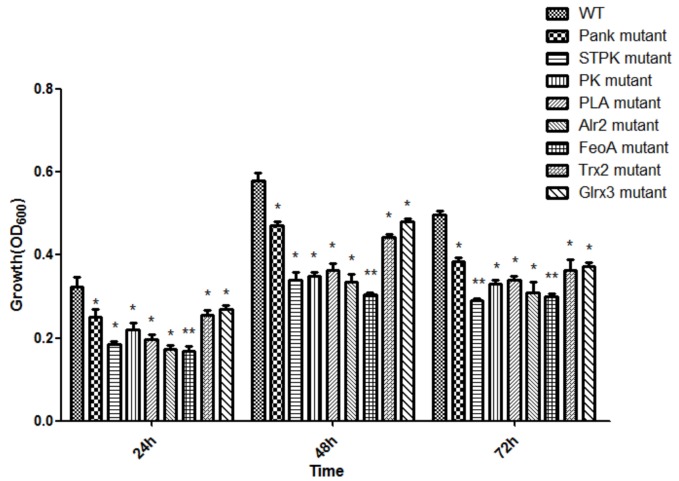
Biofilm formation of *C. malonaticus* wild strain (WT) and mutants under 1.0 mM H_2_0_2_ oxidative stress. ^∗^Significant difference about growth under 1.0 mM H_2_0_2_ with WT strain (*p* < 0.05), ^∗∗^significant difference about growth under 1.0 mM H_2_0_2_ with WT strain (*p* < 0.01).

**FIGURE 3:**
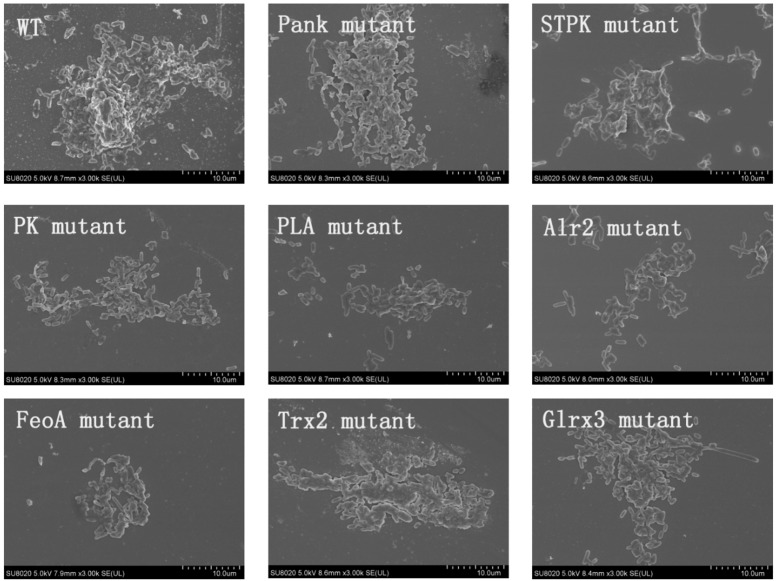
Biofilm formation of *C. malonaticus* wild strain (WT) and mutants at 48 h under 1.0 mM H_2_0_2_ using SEM.

**FIGURE 4:**
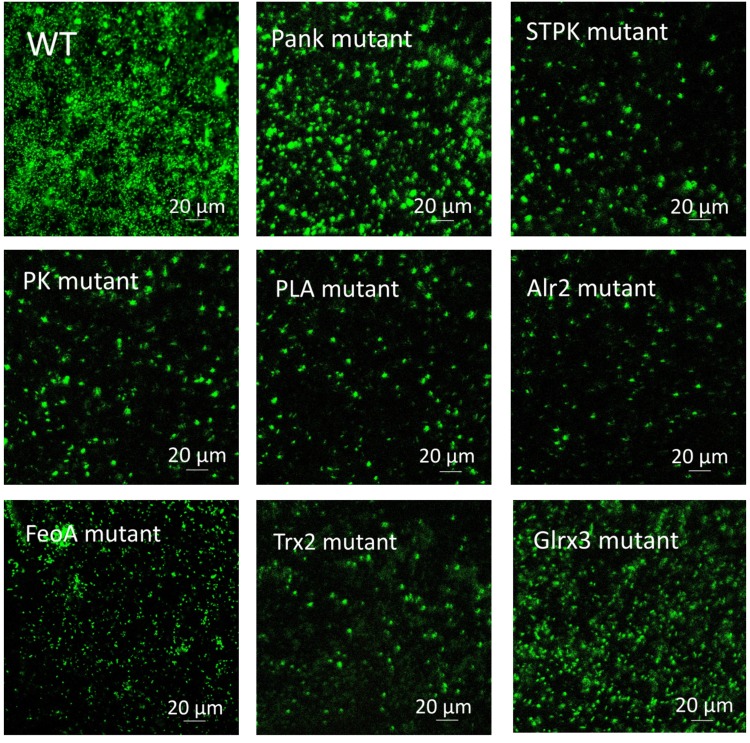
Biofilm formation of *C. malonaticus* wild strain (WT) and mutants at 48 h under 1.0 mM H_2_0_2_ using CLSM.

Based on analysis of biofilms using CVS, the strong biofilm-formatting abilities among eight mutants and WT were observed, and biofilms of eight mutants significantly decreased at 48 h compared with that of wild type (WT) shown in Figure 2. Furthermore, the detection of spatial structure of biofilms was confirmed using SEM (Figure 3), and the mature biofilms were formed at 48 h among mutants and WT. From Figure 4, the viable cells and exopolysaccharides (blue) were more predominant at 48 h. Here, inactivity of eight factors caused weak biofilms compared with that of WT under oxidative stress, and a positive relationship between biofilm formation and oxidative tolerance was observed. Hartmann et al. (2010) demonstrated that cellulose and flagella facilitated biofilm formation in *C. sakazakii*. Using comparative proteomics analysis, genes including *LuxS* and *TolB* were found to contribute to biofilm formation in *Cronobacter* strains (Ye et al., 2016). In addition, the *deoB*, *adh*, and *nlpD* were involved in biofilm formation in *C. sakazakii* (Du et al., 2012). In addition, environmental conditions such as temperature and pH also greatly affected biofilm formation in *C. sakazakii* strains (Jung et al., 2013; Ye et al., 2015). In *Haemophilus influenzae*, expression abundance of peroxiredoxin–glutaredoxin increased in biofilms compared to planktonic cells (Gallaher et al., 2006). Similarly, thioredoxin, peroxidase, and thioredoxin were upregulated in biofilms in *Candida albicans* (Seneviratne et al., 2008). The biofilm formation in *trxB* mutant of *Neisseria gonorrhoeae* on human cervical epithelial cells was greatly reduced compared with wild-type strain (Potter et al., 2009). In *S. typhimurium*, the Feo system has been found to play important roles in colonization of the mouse intestine (Tsolis et al., 1996). Jiang et al. (2015) found that hydrolase and pantothenate kinase were detected in the *Streptococcus mutans* 593 biofilm only, indicating that pantothenate kinase was involved in the biofilm formation in *S. mutans* 593. The high pyruvate kinase activity in *S. mutans* contributed to the cariogenic biofilm formation in caries patents (Krzyściak et al., 2017). Pyruvate kinase activity in *Staphylococcus aureus* was regulated by serine/threonine protein kinase, which favors biofilm formation (Vasu et al., 2015). Serine/Threonine kinases (STPKs) have been implicated in biofilm formation of *Bacillus subtilis* (Madec et al., 2002). Ser/Thr protein kinase PrkC mediates biofilm formation in *B. anthracis* by regulation of GroEL activity (Arora et al., 2017). Phospholipases (PLs) are considered important factors for *C. parapsilosis* adherence, tissue penetration, and host invasion (Junior et al., 2011). Meanwhile, the germination, adherence, biofilm formation, phospholipase and proteinase production were considered the virulence factors in *Candida albicans* (Larkin et al., 2017).

## Conclusion

In summary, the factors involved in tolerance to oxidative stress in *C. malonaticus* were identified including Trx2, Grx3, Pank, STPK, PK, PLA, FeoA, and Alr2. A positive relationship between biofilm-forming ability and oxidative tolerance was also observed, which might indicated that biofilm formation was related with environmental stress. The findings here provide valuable information for deeply understanding molecular mechanism about tolerance to oxidative stress.

## Author Contributions

XZ carried out the experiments and analyzed the data. MZ carried out the experiments and analyzed the data. LT analyzed the data and carried out the experiments. DO carried out the partial experiments. YW analyzed the data. JZ modified the manuscript. QW and YY designed and modified the manuscript.

## Conflict of Interest Statement

The authors declare that the research was conducted in the absence of any commercial or financial relationships that could be construed as a potential conflict of interest.
